# Usability of Health Information Websites Designed for Adolescents: Systematic Review, Neurodevelopmental Model, and Design Brief

**DOI:** 10.2196/11584

**Published:** 2019-04-23

**Authors:** Gurpreet Kaur Reen, Linden Muirhead, Dawn Wendy Langdon

**Affiliations:** 1 Royal Holloway, University of London Egham United Kingdom; 2 University of Oxford Oxford United Kingdom; 3 MS Trust Letchworth Garden City United Kingdom

**Keywords:** adolescents, health communication, internet, information seeking behavior, usability, systematic review

## Abstract

**Background:**

Adolescence is a unique developmental period characterized by biological, social, and cognitive changes, as well as an interest in managing one’s own health care. Many adolescents use the internet to seek health care information. However, young people face barriers before they can understand and apply the health information that they access on the web. It is essential that usability of adolescent health websites on the internet is improved to help adolescents overcome these barriers and allow them to engage successfully with web-based health care content.

**Objective:**

The aim of this review was to synthesize the usability of specific health information websites. These findings were mapped onto the adolescent neurodevelopmental profile, and a design brief based on the findings was developed to tailor future websites for specific adolescent requirements.

**Methods:**

A systematic search conducted using PubMed, PsycINFO, and Education Resources Information Center (ERIC) identified 25 studies that assessed the usability of health information websites. Adolescent feedback was collected by a mixture of surveys, focus groups, interviews, and think-aloud procedures.

**Results:**

A majority of the information websites were developed for specific health issues that may be relevant to adolescents. The most preferred website features were interactive content such as games and quizzes, as well as videos, images, audio clips, and animations. Participants also preferred communicating with other adolescents with similar conditions or learning about their experience through real stories and testimonials. Adolescents found it difficult to use health information websites if they contained too much text, were too cluttered, or had features that made it difficult to access. The findings are considered in the context of adolescent social processes, low tolerance of delayed gratification, and attraction to novelty and mapped onto a neurodevelopmental model of adolescence.

**Conclusions:**

Young people’s feedback can determine usability and content that make a health information website easy or informative to use. Neurodevelopmental profiles and the users’ specific preferences and skills should be addressed in future development of health information websites for adolescents.

## Introduction

### Background

Adolescence is a significant period of development between childhood and adulthood (occurring approximately between the ages of 10 and 22 years) that is generally characterized by changes in social skills, cognitive capabilities, and a keen interest in one’s own health care and well-being [1,2]. To better understand and manage their health, adolescents often look for information about specific health areas including sensitive topics such as sexual and mental health, as well as general guidance on health-related matters such as physical fitness and diet [3,4]. The internet is an appealing and valuable source for adolescents to seek this information [4-8]. A survey of over 1000 young people found that 84% of adolescents reported having used the internet at least once in their lifetime to access health information [3]. The advantages of the internet as a tool to find health information include easy access to a wide range of health topics, the ability to reach a large audience, and the ease with which adolescents can search for sensitive information anonymously [3,9]. Nevertheless, adolescents do not always find it easy to access the health information they find on the web.

Barriers to accessing health information often stem from low health literacy skills in the younger population. In the context of web-based information seeking, low health literacy in younger people can result in difficulty to either locate relevant health information on the internet, evaluate the credibility of the information they find, or apply this information to their personal life [4,6,7]. For instance, a large systematic review identified that adolescents find it difficult to create appropriate search strings when using search engines to find health information on the web [6,10]. Particularly, adolescents are likely to misspell medical terms resulting in a search that is irrelevant and confusing [7,10]. With regard to credibility, adolescents are keen to access information from reputable sources and generally avoid sites that are overtly commercial with excessive advertisements and links [6]. Furthermore, they may also stop their search as soon as they find the health information they need without comparing it with other possible sources [6,10]. Adolescents are thus not always equipped with appropriate skills and strategies to access credible web-based health information.

To overcome such barriers, accurate and evidence-based health information should be provided in a website customized for the younger population so that adolescents do not have to use unreliable internet sources [11]. In addition to content, the usability of these websites should be designed for the younger audience to ensure that adolescents are able to engage with the health care content [12]. For instance, a website that is poorly organized and presented can prevent young people from understanding and sometimes even retrieving the health information they require [10]. Improving the usability of websites with health information (referred now on as health information websites) can be a simple solution to the barriers faced by young people on the web. One method of improving usability is to incorporate the views and preferences of the users during the development of health information websites. By employing this user-centered approach, websites can be specifically tailored to the adolescent population, which can increase the likelihood that young people will access and regularly use the website content to better manage their health care [12-14].

Furthermore, it is now understood that adolescence is characterized by specific neurobiological, cognitive, and social development. Typical adolescent development includes, for example, a peak in sensation seeking and poor tolerance of delayed gratification, which are likely to affect how adolescents experience health information websites. The increased sensation seeking leads to risk taking, which has been explained by how differently the adolescent brain processes affective and social information, both likely to be found in health information websites [15,16].

Despite a large number of adolescent health information websites available, only a few have evaluated their usability with feedback from the younger population. A recent study reviewed general web-based health–searching behavior in adolescents [17]. However, there has yet to be a review that synthesizes adolescent feedback on the usability of specific health information websites.

### Objectives

The purpose of this review was to determine adolescent preferences for the usability of specific health information websites and highlight the difficulties adolescents face when attempting to access the content on these sites. It is expected that this review will help inform the development of new adolescent health information websites, as well as allow existing adolescent health information websites to be adapted to their target audience. The findings of this review were considered in the context of the neurodevelopmental profile of adolescence including social processes, low tolerance of delayed gratification, and attraction to novelty.

## Methods

The Preferred Reporting Items for Systematic Reviews and Meta-Analyses recommendations were used as guidelines for the presentation of this review [18]. A protocol for this review has not been previously published or registered.

### Systematic Literature Search

A systematic literature search was conducted in April 2018 using PubMed, PsycINFO, and Education Resources Information Center (ERIC). Uniform search terms were developed and used with all 3 databases (see Textbox 1).

### Eligibility Criteria

Studies were included in this review if the following criteria were met: peer-reviewed original studies in English, studies with some adolescent participants aged between 13 and 17 years, studies about a health information website for any health topic, and studies that conducted some form of usability testing of the website from the perspective of adolescents. Both clinical and nonclinical adolescent populations were included into this review. Health information websites were defined as health websites which predominantly provide information about a specific health topic or provide general health guidance. The definition of health websites used in this review was adopted from the study by Sillence et al [19]. All types of study designs from the year 2000 were included if the inclusion criteria were met. The year 2000 was chosen so that a reasonable number of websites could be included in the review, although remaining moderately current.

**Search terms to shortlist studies.**

adolescents OR young people OR teen OR young adult OR youth

AND

health OR nutrition OR sex OR illness OR medical condition OR disease OR alcohol OR tobacco OR physical activity OR diabetes OR cancer OR weight

AND

website OR online OR internet OR web 2.0

AND

evaluation OR views OR opinion OR survey OR usability OR feasibility OR user feedback

Studies were excluded if they only included participants aged older than 18 years or younger than 13 years. Studies that evaluated any other form of web-based health content were excluded (eg, health information presented through social media platforms, web-based courses, short-term education modules, support groups, and tools to collect patient data). Studies were also excluded if they evaluated usability of health websites that did not predominantly provide information about health topics. Studies were also excluded if they mostly focused on changing adolescent health behavior. It was expected that these studies evaluate usability features that are unique to websites designed to change health behavior but are not usually present in typical health information websites (eg, tools to monitor health behavior, feedback on progress, record of goals and plans, tailored content, review of previous health behavior, and messages of encouragement and motivation). Studies that only described a health information website, did not evaluate usability from the feedback of its users, or only described a protocol for a study were further excluded. Studies evaluating a nonhealth–related information website were also excluded from this review.

All titles and abstracts were screened. Studies that were deemed suitable from additional reference checking or searching through journals were also included. After initial screening, 302 studies were considered for eligibility and full texts were subsequently accessed.

### Data Extraction

**Figure 1:**
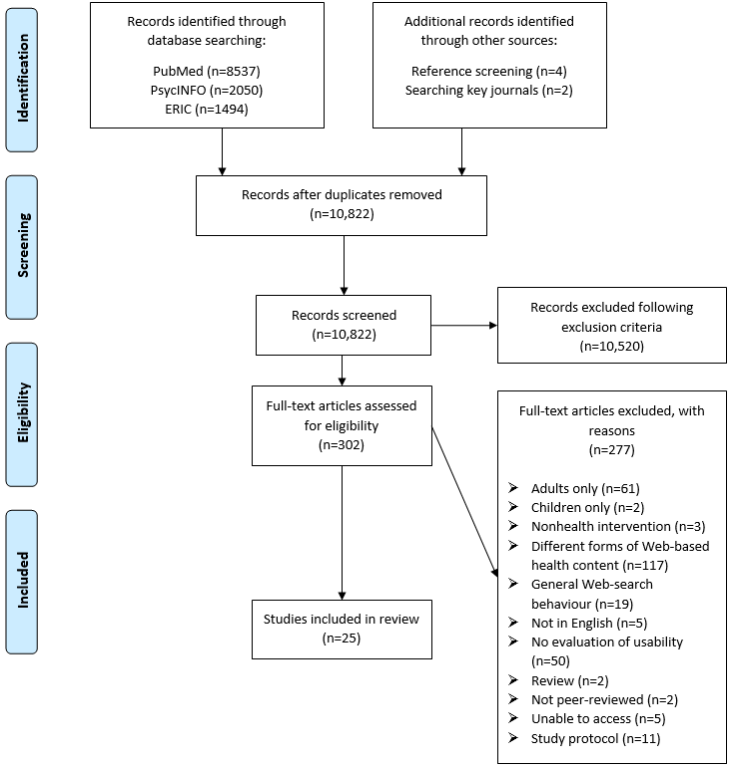
Preferred Reporting Items for Systematic Reviews and Meta-Analyses flow chart for selection process of studies in systematic review. ERIC: Education Resources Information Center.

Data extraction forms were designed to extract relevant information from full texts and assess their eligibility for the final review. Extraction was initially carried out by 1 reviewer (GR) and was then verified by another (DL). Any discrepancies between extractions were resolved by discussion. After data extraction, 25 studies were included into the final review (see Figure 1).

All the studies were broadly qualitative, in the sense that they collected interview data and utilized ad hoc surveys. The narrative synthesis in this review was primarily based on the qualitative findings from these studies. Participant demographics were extracted from the shortlisted studies, comprising (where reported) age, gender, and whether participants were from the nonclinical or clinical population. Relevant information about the health information websites were also extracted, including the topic of the website and the characteristics of the website. The authors also noted whether the website was currently on the web. The method used to evaluate usability was also recorded. Any data that recorded adolescents’ feedback on the usability of the website were extracted. Data were extracted from information in texts, tables, figures, and qualitative feedback.

Results were categorized into themes based on 5 of the 8 areas important for the development of health-related websites, as highlighted by Ritterband et al [12]. These areas are relevant to the usability of websites and include appearance (eg, visual appearance, organization of information, and screen size), burdens (eg, barriers to navigation), delivery of content (eg, animations, videos, illustrations, vignettes, and testimonials), message source (eg, credibility and age-appropriateness of the website), and participation (eg, the degree of interaction by the users). Understanding the adolescents’ preferences for each of these domains could inform how each area should be tailored for the adolescent population when developing websites. The 3 remaining areas of website development were beyond the scope of this review (ie, content of the website, behavioral prescriptions or guidelines, and adapting website content per users’ assessment and feedback) [12]. Results based on each of the 5 areas were further categorized by clinical or nonclinical adolescent users, by younger (aged ≤14 years) or older adolescents (aged >14 years), and by adolescents’ gender.

### Quality Rating

The Critical Appraisal Skills Programme (CASP) tool was used to assess the quality of the studies in this review by 2 independent reviewers (GR and DL). This tool was chosen as it has been recommended for reviewers when appraising qualitative studies [20] and has previously been used in other health-related systematic reviews [21,22].

The CASP tool is a checklist comprising 10 questions that broadly assess aims and methodology, appropriateness of research design, data collection, data analysis, findings, impact of investigator, ethics and values, and implications of research [20]. Reviewers are asked to rate each question on the checklist as yes, no, or unclear. Each of the questions are followed by prompts to help reviewers consider the criteria. The checklist also encourages reviewers to record the reasons for their response to each question. CASP is considered as a tool to assess the general quality of the study but does not offer any objective reason to exclude a study from the review. Therefore, no studies were excluded from this review based on the quality rating. There were only slight discrepancies between reviewers when rating study quality, which were resolved by discussion until an agreement was reached.

## Results

### Participant Demographics

A total of 25 studies evaluating usability of health information websites for adolescents were included into this review, and a quality appraisal was done for all studies (see Multimedia Appendix 1). A total of 2621 participants were recruited across all studies, ranging from ages 11 to 25 years (see Table 1). The majority of the website users were from a nonclinical population: 79.13% (2074/2621), followed by adolescents diagnosed with diabetes: 12.28% (322/2621), juvenile idiopathic arthritis: 2.06% (54/2621), hemophilia: 1.79% (47/2621), depression: 1.60% (42/2621), cancer: 0.84% (22/2621), cystic fibrosis: 0.61% (16/2621), migraines: 0.45% (12/2621), and those that had recently undergone a kidney transplant: 0.80% (21/2621). The mean age of participants was 15.2 years. Most studies included both younger (≤14 years) and older adolescents (>14 years).

In total, 9 studies did not provide enough information to calculate mean age [5,27,28,32,34,36,37,41,46], and 1 study only provided a median age [39]. Participants were recruited from a range of sources, including middle schools, secondary schools, specialist clinics, online, youth services, those who had participated in another study, and those who had already accessed the website. One study did not specify the location of recruitment [34].

**Table 1:** Participant demographics of included studies (n=25).

Study (first author, year)	Health status	Sample size (n)	Age range (years)	Age (years), mean (SD^a^)	Gender	Recruited from
Ammerlaan, 2015 [23]	Diagnosed with juvenile idiopathic arthritis	13	17-22	20	92% females, 8% males	Outpatient clinics
Baulch, 2010 [24]	Nonclinical	67	Not specified	15.9 (1.8)	77% females, 23% males	Secondary school and a health obesity treatment program
Breakey, 2013 [25]	Diagnosed with hemophilia	18	13-18	15.5	Not specified	Hematology clinics
Breakey, 2014 [26]	Diagnosed with hemophilia	29	13-18	15.9	100% males	Hematology clinics
Coyne, 2016 [27]	Diagnosed with cystic fibrosis	16	15-25	Not specified	Not specified	Participants from previous study
Cullen, 2013 [28]	Nonclinical	211	12-17	Not specified	54% females, 46% males	Health fairs, schools, churches, community organizations, and newspaper and radio advertisements
Danielson, 2016 [29]	Nonclinical	18	13-18	16.3 (1.5)	100% females	Department of juvenile justice, high schools, youth centers, and snowball recruitment methods
Debar, 2009 [30]	Nonclinical	82	14-16	15.6 (0.6)	100% females	Participants from previous study
Donovan, 2012 [31]	Diagnosed with migraines	12	12-17	14	50% females, 50% males	Newspaper advertisements and community message board
Ercan, 2006 [32]	Nonclinical	105	13-15	Not specified	56% females, 44% males	Comprehensive school and special hospital school for children with depression and anxiety
Franck, 2007 [5]	Nonclinical	116	11-18	Not specified	Not specified	Secondary schools
Hanberger, 2013 [33]	Diagnosed with diabetes	287	Not specified	Intervention: 13.2 (3.7); Control: 13.3 (3.7)	Intervention: 52% females, 48% males; Control: 51% females, 49% males	2 pediatric clinics
Korus, 2015 [34]	Undergone kidney transplantation	21	12-17	Not specified	33% female, 67% males	Not specified
Long, 2009 [35]	Group 1: History of chronic pain; Group 2: Current diagnosis of chronic pain	Group 1: 5; Group 2: 6	Group 1: 13-17; Group 2: 12-16	Group 1: 15.8 (1.2); Group 2: 14.6 (1.6)	Group 1:60% females, 40% males; Group 2:67% females, 33% males	Multidisciplinary pediatric chronic pain clinic
McCarthy, 2012 [36]	Nonclinical	67	16-22; 72% aged between 16 and 17	Not specified	75% females, 25% males	Sexual health clinics, colleges, youth work contacts, youth websites, and friends of participants
Michaud, 2003 [37]	Nonclinical	Approximately 1394	13-18	Not specified	63% females, 37% males	Users of website
Nicholas, 2012 [38]	Diagnosed with diabetes	31	12-17	14.5	Not specified	Pediatric hospital
Nordfeldt, 2010 [39]	Diagnosed with diabetes	4	11-18	14^b^	Not specified	Clinical intervention study
Radovic, 2017 [40]	Diagnosed with depression	27 (Phase 1: 23; Phase 2: 4)	13-21	Phase 1: 16 (2.3); Phase 2: not specified	Phase 1: 78% females, 22% males; Phase 2 not specified	Phase 1: Academic adolescent medicine clinic and specialty psychiatric clinic; Phase 2: Youth advisory board
Radovic, 2018[41]	Diagnosed with depression	15	14-19	Not specified	Not specified	Clinical settings and online
Starling, 2015 [42]	Nonclinical	10	11-13	12.7	70% females, 30% males	Middle school
Stinson, 2015 [43]	Diagnosed with cancer	22	Not specified	15.2 (1.8)	41% females, 59% males	2 pediatric cancer treatment centers
Stinson, 2010a [44]	Diagnosed with juvenile idiopathic arthritis	19	Not specified	15.7 (1.5)	74% females, 26% males	2 rheumatology clinics in pediatric tertiary care centers
Stinson, 2010b [45]	Diagnosed with juvenile idiopathic arthritis	22	Not specified	14.4 (1.3)	68% females, 32% males	4 pediatric tertiary care centers
Wozney, 2015 [46]	Nonclinical	4	15-20	Not specified	50% females, 50% males	People involved in peer advocacy for mental health and illness

^a^Not all studies report SD with mean age.

^b^Median.

### Website Characteristics

The majority of studies evaluated health information websites that were designed for specific health topics for adolescents (see Table 2). These include health information websites dedicated to weight management [24], physical activity and diet [28,30], organ transplants [34], transition from pediatric to adult health care [27], diabetes [33,38,39], juvenile idiopathic arthritis [23,44,45], human papillomavirus [42], anxiety [46], hemophilia [25,26], HIV prevention [29], depression [40,41], chronic pain [35], migraines [31], and cancer [43]. Health information websites that were developed to give general information to adolescents included a combination of health topics such as mental health, diet, drugs and alcohol, contraception, and sleep patterns. Furthermore, 4 health information websites were described by more than 1 study [25,26,33,39-41,44,45]. Some websites were already live on the internet before usability was fully evaluated [5,27,32,33,37,39], and several health information websites were still found to be on the web at the time of this review [23,25-27,29,34,37,40,41,44,45] (see Multimedia Appendix 2). One study did not provide the name of the health information website being evaluated [24].

### User Feedback

Studies conducted with a clinical adolescent population used several different methods to collect usability feedback. In total, 2 studies used surveys to identify whether users were likely to use and accept the website features [26,31,35,41,45]. Furthermore, 4 studies employed a think-aloud procedure by recording users’ thoughts as they used the website [25,27,34,40,44]. Studies with clinical adolescents also used interviews to record adolescents’ experience of using the website [23,25,34,40,41,43,44]. One study collected information about the most visited website features [33], whereas another study requested an essay from its users about the best and worst website usability features [39].

With the exception of a think-aloud procedure, studies with nonclinical adolescent populations also collected usability feedback from users through surveys [5,24,28-30,32,37], website visits [30,37], and interviews [5,29]. One study also conducted focus groups with nonclinical adolescent users before developing the website [36].

**Table 2:** Website characteristics and patient feedback about website usability (n=25).

Study (first author, year)	Topic of website	Website features	Method of evaluation	Positive feedback	Negative feedback
Ammerlaan, 2015 [23]	Juvenile Idiopathic Arthritis	Log-in feature, communication with a specialist, access to medical record, self-monitoring (eg, online diary)	Interviews	Preferred features: Website design, easy to read, well-targeted to young adults, videos, life stories of other patients. Features wanted: Facility to make online appointments, access to x-rays, printing forms for blood collection, opening hours of specialists. Most used tool: Access to personal medical records	Features not preferred: Log-in code too long and complex, access to medical records overwhelming, Least used tool: Communication with specialist (n=4) Self-monitoring (n=4)
Baulch, 2010 [24]	Weight management	Monitoring, reward ideas, links to additional ideas, charts to plot daily activity, color, interactive, discussion board, health professional to answer questions	Modified Technology Acceptance Model scale (12-item measure of usefulness and ease of use on 1-7 Likert scale) Survey	Perceived usefulness: (M=5.04, SD=0.98), perceived ease of use (M=5.48, SD=0.98), intention to use (M=4.82, SD=1.48). Most preferred features: Links to additional ideas (97%), charts (84%), email feedback (82%), login (79%), links to other websites (76%), phone support (61%), discussion board (57%), chat room (52%). Additional preferred features: Games, success stories, motivation quotes	Least preferred feature: Online journal (37%)
Breakey, 2013 [25]	Hemophilia	80 web pages of content, images, interactive animations, quizzes, glossary, self-management strategies	Think-aloud procedure; Interviews	Preferred features: Color, chunking of information, images, animations, interview of peers with condition	Features not preferred: Videos too dark (n=3), Venn diagrams difficult to comprehend
Breakey, 2014 [26]	Hemophilia	80 web pages of content, images, interactive animations, quizzes, glossary, self-management strategies	Survey	Preferred features: Easy to use, videos, animations, relaxation exercises	Least used feature: Internet forum
Coyne, 2016 [27]	Transition from pediatric to adult healthcare	Home page, top-tips section, 9 video testimonials, FAQs, essential reading, external links, photo gallery	Think-aloud procedure	Preferred features: Good layout, easy navigation, testimonials, short length videos (2-5 min), age-appropriate, colorful Features wanted: Downloadable information sheets, visual images of clinics and hospitals	Features not preferred: Pages too long, too much information, bright orange color, picture of young person considered cheesy, did not understand meaning of FAQ
Cullen, 2013 [28]	Physical activity and diet	Log-in feature, healthy eating calculator, goal setting, 12 short role-model video stories, Did you know section, blog, track goal progress (diary), print goal sheet	Survey	Preferred features: Healthy eating calculator (89%), Goal setting (91%), Did you know section (88%)	Least used features: Diary used 3 or more times (33%)
Danielson, 2016 [29]	HIV prevention	Subsections, videos, photos of women, interactive quizzes	Survey; Interviews	Preferred features: Videos, age-appropriate, interactive activities and quizzes, pictures of other females	Features not preferred: Videos too long, mixed reviews on navigation (some found it easy to navigate and some found it difficult to navigate)
Debar, 2009 [30]	Physical activity and diet	Log-in feature, bulletin board to communicate with peers and staff, special form to ask questions to staff for confidential reply, forum to post anonymous questions, handouts, links to external content, hot tips section providing short summaries, photos of staff, quizzes, options to win prizes, incentive points, My progress page	Survey; Website visits	Most preferred features: Incentive points system (50%), Learning new information (37%). Most visited pages: Fun stuff such as quizzes (35%), Social networking features (29%), Scrapbook (24%)	Least preferred features: Information not regularly updated (30%). Least visited pages: Hot tips summaries (8%), additional resources (4%)
Donovan, 2012 [31]	Migraines	Quizzes, audio and video-based tools, social networking, virtual toolbox of coping strategies, headache diary	Survey	Features preferred: Ask an expert feature, video-based content. Features wanted: Library of content	—^a^
Ercan, 2006 [32]	General Includes: mental health, eating problems, drugs and alcohol	interactive stories, cartoons about depression, games	Survey	Preferred features: Graphics/pictures (86.6%), site customization (85.1%), games (76.9%), interactive stories (83.1%)	—
Franck, 2007 [5]	General; Includes: body, chronic conditions, disabilities, tests, treatments	Sections dedicated to children, teens and families section	Analysis of 30 min website navigation; Survey; Informal discussion	Navigation: 45% went to older adolescent section before going to section suitable for age Preferred features: Pictures, games and animations, real stories. Features wanted: Positive recovery stories	Features not preferred: Gender imbalanced pages, too much text in children section, the cartoon characters used, real stories section too negative, older adolescent page considered dull and boring, disagreement for pages too crowded or too plain
Hanberger, 2013 [33]	Diabetes	Social networking, discussion board, local practitioners’ details, information about local activities, new research, questions and answers, photos of staff, education videos, date of last update and people who wrote webpage	Website visits	Most visited pages: Home page (10%), stories section (5.3%), blogs (7.5%), questions and answers (1.8%), videos (2.7%), discussion board (1.2%)	Least visited page: External links (0%)
Korus, 2015 [34]	Transplant	Videos, colorful	Think-aloud procedure; Semistructured interviews	Preferred features: Visually-appealing with lots of color, video testimonials Features wanted: Hyperlinks to other pages within module, a search box, drop-down menus, more color, more pictures and graphics, a discussion forum, music, interactive qualities	Features not preferred: Mixed reviews about navigation (some found it easy to navigate and others found it difficult to navigate)
Long, 2009 [35]	Chronic pain	Video interviews of peers with condition, relaxation audio clips, 200 content pages with graphics, log-in, goal setting, interactive, questions and answers	Stage 1: Survey for users with past history of chronic pain (1-5 Likert scale). Stage 2: Survey for users with current chronic pain; 1-5 Likert scale)	Stage 1: Perceived ease of use: (M=4.40, SD=0.55). Stage 2 Perceived ease of use: (Mean=4.50, SD=0.64); Preferred features: Easy to navigate, video, audio, personalization	Stage 1: Features not preferred: Difficult to understand images, lengthy content. Stage 2: Features not preferred: Some images, long load times
McCarthy, 2012 [36]	Sexual health	Interactive quizzes, short interactive activities, peer-to-peer exchange of views (website deigned after user feedback)	Focus groups	Preferred features: Social interaction, anonymous, videos about peers discussing real stories, commenting on videos, dramatic story format, images of people, images of scenarios, images about specific sexual health issues, easy to understand website logo, clear and memorable website name, weekly update, interactive activities	Features not preferred: Too much text, disagreement on color (some users preferred bold colors and others preferred neutral tones)
Michaud, 2003 [37]	General; Includes: drug, alcohol, contraception, sleep patterns	Emailed questions answered by professionals, addresses of professional institutions, list of previously asked questions	Survey; Website visits	Preferred features: Structured answers to questions, list of other questions, addresses of other institutions. Most visited page: Questions and answers (82%)	—
Nicholas, 2012 [38]	Diabetes	Not specified	Interviews	Preferred features: Discussion topics on forum, anonymity Features wanted: Personalization, instant-messaging	—
Nordfeldt, 2010 [39]	Diabetes	Social networking, discussion board, local practitioners’ details, information about local activities, new research, questions and answers, photos of staff, educational videos, date of last update and people who wrote webpage	Qualitative essays	Preferred features: Facts, simple layout, easy to use and log-in	Features not preferred: Difficult chatting feature, difficult log-in as password too hard to recover
Radovic, 2017 [40]	Depression	Blog posts, Links to external resources, personalization, questions posed for user discussion	Phase 1: interviews Phase 2: Think-aloud procedure and System Usability Scale (1-5 Likert scale)	Phase 1: Most preferred features: online peer support, blogs, moderator to avoid sharing incorrect information with other peers, anonymity. Phase 2: 100% completed all think-aloud tasks; Usability score mean=4.5 (SD=.31)	Phase 2: Least preferred feature: Wiki tool to contribute to a story
Radovic, 2018 [41]	Depression	Blog posts, links to external resources, personalization, questions posed for user discussion	System usability scale (1-5 Likert scale); Survey; Interview	Preferred features: Age appropriate format, positive atmosphere, interactive, regular new content, positive stories, anonymity. Features wanted: Social interaction, interactive. Rating of user friendliness: 50% users thought user friendly of site was good	Features not preferred: Structured communication on forum, log-in
Starling, 2015 [42]	Human papillomavirus	Interactive quiz show game, texting stimulation, FAQ section	Analysis of navigation System usability scale (Rating of user friendliness, 1-7 Likert scale); Survey	Preferred features: Easy to use, website functions. Rating of user friendliness: (M=6.0). Features wanted: Clear title	Features not preferred: Sound levels of videos too high
Stinson, 2010a [44]	Juvenile Idiopathic Arthritis	310 content pages, animations, images, videos of peers with medical condition, written stories, discussion boards, surveys, quizzes, glossary of medical terms, relaxation audio clips, visual imagery audio clips, printable PDF information forms for teachers, journal for symptom tracking and weekly goals, ask the expert features, personalization	Analysis of 30-45 min navigation; Think-aloud Semistructured interviews	Preferred features: Animations, chunking of text, layout, simple website, up-to-date, age-appropriate, glossary of medical terms, ask the expert, pdf forms, audio clips, journal, discussion boards. Features wanted: Chunking of texts using graphics and animations, labels on medical diagrams, important information on top of page	Navigation errors: Overall navigation errors (10%), Presentation error (26%), Control usage of animations and videos error (42%). Features not preferred: Teenager looking sad, control buttons on animations and videos not easy to see
Stinson, 2010b [45]	Juvenile Idiopathic Arthritis	310 content pages, animations, images, videos of peer with medical condition, written stories, discussion boards, surveys, quizzes, glossary of medical terms, relaxation audio clips, visual imagery audio clips, printable PDF information forms for teachers, journal for symptom tracking and weekly goals, ask the expert features, personalization	Survey	Preferred features: Easy to use, video, relaxation audio guide, visual imagery audio guide, personalization	—
Stinson, 2015 [43]	Cancer	200 contents pages, animations, images, videos, discussion boards, surveys and interactive forms, log-in required	Analysis of 30-40 min navigation; Think-aloud Semistructured interviews	Preferred features: Color scheme, sections and subsections making it easy to locate information, bright colors, graffiti-style wallpaper, simple words with not too much jargon, videos from experts, glossary of terms, interactive components, video clips of other adolescents. Features wanted: Font and appearance to take up more room, search bar at the top of the page	Features not preferred: Too much blank 'white' space
Wozney, 2015 [46]	Anxiety	Personalization (Email reminders, prepopulating answers, notifications), videos, log-in, check-in & check-out, homework activities	Interviews	Preferred features: Introductory animation video, personalization, comic book style, videos, images, graphics, animations, logos. Features wanted: More videos and images needed	Features not preferred: Slow page loading, no chunking, not visually pleasing

^a^Not applicable.

#### Visual Appearance

Websites that used bright colors were appreciated by adolescents with medical conditions [25,27,34,43], with users of 2 websites stating that they would like more color [27,34]. Color was preferred by both the younger [25,34] and older adolescent population [25,27,34,43], as well as by both genders [34,43]. One study did not evaluate the specific visual features of a website, but adolescents reported that they favored the overall appearance and design of the website [23]. Adolescent users also stated that they did not like a lot of blank space on the website’s page [43] and preferred a website with a simple layout [44]. These users were mostly older male and female adolescents.

Similar to the clinical population, nonclinical adolescents did not like websites that looked too dull or boring [5] or had a lot of blank space on the page [5]. Websites with excessive text were also not considered visually appealing [5,36,46] and in fact led to users in 1 study to state that they did not like the overall visual appearance and website design [46]. This was true for both male and female adolescent groups [36,46], as well as younger and older adolescents [5,36,46].

#### Navigation Burden

A total of 10 studies recorded adolescents’ navigation of health information websites. Adolescents diagnosed with medical conditions across 6 studies reported that they were easily able to navigate the website [27,35,39,40,44,45]. Even younger adolescent groups found the website easy to navigate [39,40], although a difficult log-in procedure was not appreciated [39]. In 1 study, users stated that they did not like the log-in procedure at all [41]. Users in another study offered mixed opinions about the navigation of the website; some users found the website easy to navigate, whereas other users found the website difficult to navigate [34]. These groups of users did not differ in age or gender compared with adolescents who felt generally positive about website navigation. With regard to improving navigation, adolescents suggested that websites should have a search bar [34,43], drop-down menus [34], information at the top of the page [44], and hyperlinks within the text, which link to other pages on the website [34].

Similar to the clinical population, nonclinical adolescents in 2 studies found websites easy to navigate [24,42], and users in 1 study differed in their opinions regarding navigation [29]. The only difference between users who found navigation easy and those with mixed opinions was that the latter group of adolescents were all females [29]. Specifically, users did not appreciate slow loading pages or a difficult log-in procedure when navigating the website [23,39]. In 1 study, users reporting difficulties logging in to the website actually used the website less compared with users who did not report these difficulties [23]. Adolescents in these studies were of all ages and mostly female [23,39].

#### Delivery of Content

Health content was delivered in a variety of ways across websites. Participants diagnosed with medical conditions strongly expressed their preference for content delivered using videos [23,26,27,31,33-35,43,45], images [25,34], audio clips [35,44,45], and animations [25,26,44]. However, adolescents also wanted videos to be clearly visible [26], controls on videos and animations to be clearly displayed [44], “cheesy” images to be removed [27], and all content delivery to be accessible and easy to comprehend [25,43]. In fact, users did not like images that were too difficult to understand [35] and stated that medical images on the website should have labels to improve clarity [44]. Adolescents also stated that they wanted to see more images of the clinics and hospitals that they will be attending [27]. Adolescents of both genders and all ages appreciated content delivery in this manner. They also strongly preferred information that was delivered through their peers. Adolescents really liked seeing real stories and testimonials from other adolescents who had the same health issues [25,27,34,41,43], especially when presented in a video format [27,34,43], and if the stories were positive in nature [41]. Adolescents also liked content that was generated by other users, such as blogs [33,40]. Other website features that delivered content and were preferred by clinical adolescent users included graffiti-style wallpaper [43], personalization of the website [35], subsections and chunking of text [25,43,44], glossary of medical terms [44], large number of facts [39], and relaxation exercises [26]. When some of these changes were made to 1 website, adolescents reported that they found the website much more appealing and expressed their intention to use the website in the future [25]. Despite preferring a range of features, adolescents also wanted websites to be simple and not too cluttered [39,43,44]. All clinical adolescent users in these studies were mixed in terms of gender and age range.

Similar patterns were observed in studies with nonclinical populations. Adolescents also expressed their preference for website content delivered using videos [29,36,46], animations [5], and images [5,24,29,32,36,46]. Specifically, images that presented graphs, charts, or logos; images of other adolescents; or images about the health issue were appreciated by users [24,29,32,36,46]. However, adolescents did not like videos that were too long [42], videos where sound could not be easily controlled [42], images that showed unlikeable cartoon characters [5], or graphics that were too difficult to comprehend [46]. Similar to the clinical adolescent group, nonclinical adolescent users also liked real stories and testimonials from their peers and from those with similar health issues [5,32,36,37]. However, adolescents also stated that although the real stories section was a good feature, these stories and testimonials needed to be more positive and show adolescents successfully managing their health issues [5]. Other website features that were preferred by nonclinical adolescent users included information about other institutions [37], links to other websites and ideas [24], clear logo and website name [36,46], and a comic book style [46]. All nonclinical adolescent users in these studies were also mixed in terms of gender and age range.

#### Message Source

The message source of the website and how this was perceived was explored in only a few studies in this review. Clinical adolescents in 3 studies reported that they found the website age-appropriate [27,41,44]. However, adolescents in these studies were all aged 14 years or above, and it is not possible to determine whether younger adolescents would also find the website to be age-appropriate.

Nonclinical adolescent users in 1 study also considered the website to be age-appropriate [29]. Adolescents in this study were aged between 13 and 18 years, indicating that the website was suitable for all adolescent age groups [29]. Other users also offered suggestions to improve the message source of websites. Adolescent users of 2 websites felt that the logo and website name should be clearer and more suitable to the target audience [36,46]. Adolescents in these studies were mixed in gender but were all in the older adolescent group. In 1 study, adolescents also noted that the webpages were somewhat gender imbalanced (ie, too “girly” or too much information about what boys like) and needed to be adapted for all its users [5]. However, this study did not specify the number of males and females that were evaluated.

#### Participation

Many websites in this review used interactive features that were generally favored by users of these websites. Specific interactive features that were preferred by adolescents with a clinical condition include games and quizzes [25,43], a journal feature [44], and the ability to personalize the website [44]. These adolescents ranged from the young to old and included both males and females [25,43]. One study added more interactive features to the website following usability testing, and users expressed their intention to use the website in the future following the changes [25]. Only 1 website let its older clinical users access personal medical records, a feature that was preferred and used regularly, although some found this too overwhelming [23]. Participants also appreciated health information websites that allowed them to interact with health professionals and their peers. For instance, clinically diagnosed adolescents liked it when health professionals provided answers to the questions that they had posed, particularly when the answers were structured in an accessible manner [31,44] or delivered in a video format [43]. However, communicating with a health professional was the least used website feature in 1 study [23]. Social networking with other peers was another interactive feature favored by website users in 3 studies [33,38,40]. These included social networking using online peer forums [40] and discussion boards [33,38,44]. In 1 study, adolescents stated that they wanted more social interaction in the website [41]. All these studies included adolescents across different ages and genders. Clinical adolescents in only 1 study did not use the social networking feature [26]. These adolescents were all male and ranged from the young to old. However, the authors noted that this was likely because adolescents could not navigate the social networking feature and it did not reflect the adolescents' preference for the feature itself [26]. Despite the general preference for social networking, adolescents with medical conditions wanted anonymity during social interaction [38,41] and did not show much inclination for text information that they could edit, such as wiki tools or the online journal feature on websites [40]. Participants also disliked if it was too difficult to participate in the interactive features of the website such as if the chatting feature was too difficult to use [39]. This negative feedback was not specific to a particular age range or gender.

The nonclinical adolescent population also expressed their preferences for more interactive features. These include games and quizzes [5,24,29,30,32,36,42], ability to set goals [28], ability to customize the website [32,46], a scrapbook feature [30], an incentive point-based system [30], and an interactive demonstration of health information [29]. Adolescents in these studies were generally mixed in age and gender. Similar to the clinical population, nonclinical adolescents preferred to interact with health professionals and their peers. For instance, participants liked it when health professionals provided structured and accessible answers to the questions that they had posed [37]. Social networking with other peers was another popular feature [24,30,36]. This includes social networking using discussion boards [24], chat rooms [24], and phone support [24]. Nonclinical adolescent users across these websites were of all ages and gender. One study found that participants did not like to use the online journal feature on the website [28]. This study did not differ in terms of users’ age or gender compared with studies that only had positive feedback about the social networking feature.

## Discussion

### Principal Findings

With the advent of health information websites available on the internet, adolescents have access to a large array of content to manage their health and well-being [1,3]. However, adolescents face many challenges when obtaining and appraising web-based health information [6,10,47]. To overcome these barriers, it is essential that health information websites are suitably targeted to the adolescent population. This not only means providing relevant health-related content to young people but also improving the usability of websites so that young people are better able to access web-based health care information [12]. This systematic review was the first to synthesize the preferences and difficulties that adolescents face when using specific health information websites. The findings of this review can help tailor new and existing health care websites designed for adolescents and increase the likelihood that adolescents will successfully be able to access the health care information they require to better manage their health.

A total of 25 health care websites were identified by this review, the majority of which were designed to provide information about specific health topics. Health care content on these websites were mostly delivered using interactive features (eg, quizzes and games), videos, audio clips, animations and illustrations, and with some form of social networking. All these features were preferred by adolescents engaging with these websites, irrespective of medical diagnosis, age, and gender [5,23-32,34-36,38,40-46]. These features should be seriously considered when developing future health information websites for younger people. For instance, using interactive and digital content such as videos, images, and graphics, may help to chunk the text on a website’s page, as suggested by the users of 1 website [44]. This is especially important as adolescents do not appreciate information presented in a continuous stream of text [5,35,36,46]. Nevertheless, any features presented along with the text should still serve a purpose and not make the webpage too cluttered, as this can prevent adolescents from browsing the website altogether [10]. Illustrations, videos, and animations should also be easy to access and understand to prevent adolescents from getting distracted [25,35,44]. Another commonly preferred feature on health care websites was stories and testimonials from other young people sharing their experience with the health issue [5,25,27,32,34,36,41,43,44]. Adolescents felt that they were able to resonate with other people’s experiences more so than general facts about the health topic but preferred the experiences to be positive in nature. Adding this feature may also help to improve adolescents’ motivation to continue engaging with the website, perhaps because they identify with the health issue presented in the story [12]. Future health care websites may consider combining testimonials and stories with other favored features to improve usability of web-based health care information for this population.

The findings from this review support the view that an archetypical “digital native” with an easy, intuitive facility for media may not be broadly representative of younger people [48] and that there is still a requirement in this group for improved usability of websites [6,10]. In addition, although young people (in the United States at least) spend 7.5 hours per day every day of the week engaged with media, one-third of this time involves juggling several media simultaneously. Multitasking disrupts concurrent learning (both in the classroom and during homework) and influences cognitive function on single tasks. This is even true of background television [49]. Despite this, health information websites are usually designed with the adolescent users’ undivided attention in mind.

Adolescence is also a significant and unique period of development. The cognitive and socioaffective developments in adolescence are linked to significant dynamic changes in the brain structure and function [2,50], and the trajectories of structural development are remarkably consistent [51]. It is interesting to examine how far the feedback of adolescents regarding health information websites reflects their neurodevelopmental stage. White matter connections increase during this period, facilitating communication within the cerebral cortex. These connections eventually evolve to adult patterns, which is linked to growing behavioral control and better ability to wait for a reward (prefrontal connections to the subcortical striatum) [52]. These connections not yet being fully formed in adolescents could explain why adolescents have high intolerance of slow loading pages [35,41,46] and are likely to stop searching a website if they are unable to retrieve information quickly [10]. The feedback that real stories from other young people needed to be more positive and real stories should feature other adolescents who had successfully managed their health condition [5,41,44] could be linked to adolescents’ increased sensitivity to happy faces compared with adults [53] who presumably designed the website. Brain regions that relate to social function (medial prefrontal cortex, superior temporal cortex, and temporal parietal junction) evolve quickly in adolescence [54,55], which may also explain the preference for social networking [24,30,33,36,38,40,41,44]. Nevertheless, no health information websites explicitly profess to have tailored their design for the cognitive skill profile of adolescents. However, some models of health literacy consider children’s age-specific cognitive development [8], and there has been some consideration of how media use more generally relates to adolescent neural development [56]. Neurocognitive studies may be a way of assessing the efficacy of health messages delivered to the younger population [57]. Taking account of these and other related findings, we have constructed a neurodevelopmental model and corresponding design brief for adolescent health information websites (Table 3).

**Table 3:** Mapping of adolescents’ feedback on health information websites, their neurodevelopmental underpinnings, and design indicators.

Adolescents’ feedback	Specifics	Psychological profile	Neurodevelopmental underpinnings	Recommendations from adolescents	Design indicators
Visual appearance	Bright colors ✓^a^; Blank space x^b^; Too much text x	Novelty-seeking, high susceptibility to rewards [57]	Hypersensitivity of reward regions. There is an adolescent peak in reward-related ventral striatum, specifically nucleus accumbens, activity [58]	More color	Balance of white space to avoid clutter; clear correlation between input and output (reward value); consider accessibility of color options—target Web Content Accessibility Guidelines 2.0 conformance level AA; use images only where they add value; chunking pages into smaller bite-size bits
Navigation burden	Search features ✓; Use of hyperlinks ✓; Slow loading pages x; Difficult log-in x	Low tolerance for delayed gratification [59]	Delay tolerance positively correlates with the resting state functional connectivity between the dorsal anterior cingulate cortex and the left dorsolateral prefrontal cortex, a critical functional circuit in the cognitive control network [59]	Search bar; drop-down menus; hyperlinks to other pages	Consider weight of page, especially as images, graphics, and videos can add to slow loading; resize images to decrease weight on page; enable different ways to search and access, including effective tagging, use of synonyms, filtered and faceted search, and drop-down filters; create different ways for users to search or browse, including effective use of breadcrumbs and anchors; use language appropriate for adolescents for navigational menus; create user journeys with relevant links between sections of the site; use meaningful words to describe links; clearly display whether an in-site link or to an external site; user test log-in process; only capture data as required for fulfilment of service; clear links to privacy statements; clear wording around opt-in and opt-out permissions; 1 click log-in functions, that is, login through Google or Facebook
Delivery of content	Videos ✓; Images, graphics, and charts ✓; Audio ✓; Animation ✓	Processing narratives, emotional stimuli, self-relevance, and attention from salient stimuli [60]	Effective video health messages for young people involve responses in dorsomedial prefrontal cortex, insulae, and precuneus [61]	Sound easy to control; clearly visible; videos not too long; images or graphics not difficult to comprehend; websites that were not too cluttered	Position on page relevant to content; embed videos with uploaded image; play through YouTube, Vimeo, or dedicated video service; use of playlists to manage video length; ensure transcript available for accessibility; resize images; include meaningful alt tags; use images that add value (eg, infographics); consider use of suitable images to break up text, but must be relevant and not just decorative (especially as any image will add to page load); review how screen readers will deal with images
Delivery of content	Information from health professional ✓; Questions from adolescents ✓; Vignettes and testimonials from adolescents ✓	Conscious viewing of credentials for medical expertise and to judge credibility of web-based health information [62]. Especially sensitive to how adolescents fit into their social environment [63]	Neural substrates for social behavior overlap with neural substrates underlying physical pain. This involves the dorsal anterior cingulate, subgenual/ventral anterior cingulate cortex, right ventrolateral prefrontal cortex, medial prefrontal cortex, posterior cingulate, and insula. These regions overlap, continuing to develop structurally and functionally during adolescence [62]	Structured answers from health professionals; positive testimonials from other adolescents	Clear acknowledgment of expertise; ensure consent for any personal data disclosed; framing of adolescent testimonials
Message source	Gender imbalanced pages x; unclear logo and website name x; Age-appropriate ✓	Low tolerance for delayed gratification [59]	Delay tolerance positively correlates with the resting state functional connectivity between the dorsal anterior cingulate cortex and the left dorsolateral prefrontal cortex, a critical functional circuit in the cognitive control network [59]	Make websites age-appropriate; adapt websites to both gender	Use age-appropriate language; use of age-appropriate skins; ensure design reflects trends in adolescent behavior; recognize different age ranges with age-appropriate backgrounds, images, and personalization; consider age-based content segmentation; use colors and designs appropriate for all genders; clear positioning of website name and logo to create credibility in the brand
Participation	Quizzes ✓; Games ✓; Customized webpages ✓; Social networking ✓	Heightened attraction to novel and exciting experiences known as sensation seeking [57]; Friends are more important than at any other stage of life [64]	Rising dopaminergic activation during adolescence, which may reflect activity in the midbrain dopamine pathway ascending from the ventral tegmental region [57]	Interactive content	Break up text with interactive content; use games and quizzes to help teach health content, but must be relevant and necessary (especially as any interactive content will add to page load); polls to receive feedback from adolescents (such as polls on Twitter); allow personalization of webpages; enable peer-to-peer interaction and user-generated content; moderation to prevent trolling; manage safeguarding concerns; manage data protection concerns, access, and verification controls

^a^✓: indicates that this is a good feature.

^b^x: indicates that this is not a good feature.

In addition to neurodevelopmental changes, many adolescent clinical groups have particular cognitive profiles associated with their diagnosis, and health information websites should attempt to tailor to these. For example, adolescents with autism have specific challenges processing emotional and social stimuli [58] and sensory reactivity [59], which develops over their lifetime. Evaluation of health information websites for people with autism and other intellectual disabilities has tended to focus on content rather than on the format and user interface [65,66]. Similarly, young people with multiple sclerosis can encounter diminished attention, processing speed, visuomotor skills, language, and general intellectual level, with a mixed picture of loss of previously acquired function and failure of continued progress to expected adult intellectual competencies [60]. Guidance for website design already exists for other clinical and carer groups (eg, [67-69]). It is important that health information websites address users’ particular needs at the design and testing stage, with suitable user group engagement.

The feedback from young users of health information websites can be mapped onto their cognitive characteristics and associated neurocognitive substrates (Table 3). This has particular and specific implications for the technical and content aspects of website design. In fact, it is likely that the content and format of a website may compete, which is also known as argument quality and message sensation value [57,61]. Salient elements of a message may attract attention to the extent of reducing adolescents’ available processing capacity to comprehend the message and remember it and its persuasiveness. It seems possible that this could be explained by a fundamental competition between emotion and cognition for neural resources because they share some neural networks [62]. This is particularly true if negative emotional images are followed by increasing cognitive demand, a sequence likely to occur in health information websites. This poses a dilemma for designers, which can only be resolved by focus groups and other usability evaluations with the appropriate client group.

In addition, the content of adolescent health information websites and how it is delivered could be tailored to connect more successfully with adolescents. Changes in processing of affective and social information make adolescents more likely to attend to short-term rewarding outcomes, whereas many health behavior decisions require consideration of long-term negative outcomes [15]. At the time of adolescence, meta-cognitive processes undergo major changes, leading to improved cognitive control in adulthood, probably as a result of maturation of association cortices (such as the prefrontal cortex, posterior parietal cortex, and superior temporal cortex [16]). The adolescent period of instability in working memory, response inhibition, and performance monitoring could be addressed in website design to support and accommodate these characteristics and ensure that health information is successfully and comfortably communicated.

It is interesting to compare our findings with health website feedback from other groups. Parents of children with attention-deficit/hyperactivity disorder (ADHD) reported on an educational website and some commented that the appearance could be more appropriate for individuals with disability and that the content could have been more suitable and engaging for adolescents with ADHD [70]. They suggested that the website was “boring”, or disengaging, especially for adolescents. Others suggested introducing games for younger children. The comments from parents relating to this adolescent population would seem to fit with the findings from this review. Furthermore, Scanlan et al [71] investigated a large group of healthy young adults (aged between 18 and 25 years, primarily female university students), slightly older on average than the population in this review. Each participant was given a vignette and asked to imagine that they had just received a depression diagnosis. They were randomized to view either a traditional health information website for all ages, which listed evidence-based depression treatment recommendations, or a website targeting those aged between 12 and 30 years, which aggregated reports from young people who lived with depression. Website design was rated to be significantly better on the site that aggregated service user accounts, but interestingly this was not related to behavioral intentions in this nonclinical sample. Treatment decisions were related to content that was perceived to be more credible and endorsed by experts, rather than other users’ accounts. This study showed that preference for website design was apparently not the main behavior driver for an older, healthy cognitively intact sample imagining they were depressed. Content credibility was a significant influence [71]. Therefore, the effect of preference for website design on behavior needs to be investigated in a younger adolescent group, for whom the content is personally relevant.

### Strengths and Limitations

A recent review also examined adolescents’ preferences for web-based health information, as well as the type of health content that adolescents seek on the web [17]. However, Park et al did not review specific health information websites but rather focused on the adolescent’s general health-seeking behavior on the web [17]. Only 1 study in this review was the same as that included by Park et al [17]. This review extends these findings of Park et al by focusing on tangible usability features of specific adolescent health information websites based on feedback from its users (eg, the type of multimedia that should be used), leading to specific recommendations. Furthermore, this study mapped the findings of the systematic review onto a neurodevelopmental model to take account of the cognitive profile of adolescents.

However, this systematic review is not without its limitations. First, this review was conducted to understand the usability of adolescent health information websites only. With recent advances in technology, several digital interventions for adolescents have been designed with other media in mind. This includes platforms such as mobile apps (eg, [63,64], social media [3], and video games [72-74]). It is possible that the usability of these platforms is different than the usability of health information websites and should be systematically reviewed in further studies. Usability features of websites that were designed to change adolescents’ health behavior were also excluded from this review. These websites are likely to have unique usability features that were not typically present in health information websites (eg, tools to monitor health behavior, feedback on progress, tailored content, messages of encouragement, and motivation). It will be worthwhile to review the usability of websites that focus on changing health behavior in future studies. Second, this review did not evaluate the content of health information websites. In addition to good usability, it is also important that the content is age-appropriate, accurate, and based on quality evidence. Reviewing the content of the health information websites was beyond the scope of this review. Third, adolescents are a heterogeneous group. However, the differences between the younger and older adolescent group, and gender was only partially addressed in this review. It was not possible to evaluate the findings based on other demographics such as socioeconomic background and ethnicity because of limited information collected in the original studies, even though this could have influenced how web-based health information is accessed and interpreted [17]. In addition, it should be noted that studies were included even if some participants were aged over 18 years or under 13 years. It is possible that by including participants beyond the age range specified, we cannot confidently generalize our findings to the age group of 13 to 17 years. However, the age range of adolescents can vary considerably across studies, with participants aged between 12 and 24 years often considered to fall in the broad category of adolescence [17]. It was felt that by excluding studies strictly based on the age range of 13 to 17 years, studies may have been excluded that were still relevant to our central focus. Finally, this review was primarily based on qualitative studies for which a narrative synthesis was considered the most suitable. However, it should be noted that a qualitative review is subject to greater analysis bias than a quantitative review.

### Conclusions

The broad insight offered by this systematic review is that adolescents have specific preferences related to the usability of health information websites. Adolescents generally prefer interactive content such as games and quizzes, as well as images, graphics, videos, and animations. These features are generally preferred if clearly presented, help separate large pages of text and do not clutter the website. Social networking features are also favored by adolescents, such as discussion boards and chat rooms. However, if health information websites are not able to provide these features, it is recommended that real stories and testimonials from other adolescents with the relevant health issue are made available on the website. Website design should take account of the preferences, skills, and neurodevelopmental profile of adolescents. If a clinical group with a specific cognitive profile is targeted, then this should also be accommodated. The findings of this review could help inform the development of more successful health information websites for adolescents.

## Appendix

Multimedia Appendix 1 Quality assessment of included studies (n=25).

Multimedia Appendix 2 Website characteristics and patient feedback about website usability (n=25).

## Abbreviations

ADHD: attention-deficit/hyperactivity disorder
CASP: Critical Appraisal Skills Programme
ERIC: Education Resources Information Center

## References

[1] Patton GC, Sawyer SM, Santelli JS, Ross DA, Afifi R, Allen NB, Arora M, Azzopardi P, Baldwin W, Bonell C, Kakuma R, Kennedy E, Mahon J, McGovern T, Mokdad AH, Patel V, Petroni S, Reavley N, Taiwo K, Waldfogel J, Wickremarathne D, Barroso C, Bhutta Z, Fatusi AO, Mattoo A, Diers J, Fang J, Ferguson J, Ssewamala F, Viner RM (2016). Our future: a Lancet commission on adolescent health and wellbeing. Lancet.

[2] Stevens MC (2016). The contributions of resting state and task-based functional connectivity studies to our understanding of adolescent brain network maturation. Neurosci Biobehav Rev.

[3] Wartella E, Rideout V, Montague H, Beaudoin-Ryan L, Lauricella A (2016). Teens, Health and Technology: A National Survey. Media Commun.

[4] Borzekowski DL (2006). Adolescents' use of the Internet: a controversial, coming-of-age resource. Adolesc Med Clin.

[5] Franck LS, Noble G (2007). Here's an idea: ask the users! Young people's views on navigation, design and content of a health information website. J Child Health Care.

[6] Freeman JL, Caldwell PH, Bennett PA, Scott KM (2018). How adolescents search for and appraise online health information: A systematic review. J Pediatr.

[7] Gray NJ, Klein JD, Noyce PR, Sesselberg TS, Cantrill JA (2005). Health information-seeking behaviour in adolescence: the place of the internet. Soc Sci Med.

[8] Bröder J, Okan O, Bauer U, Bruland D, Schlupp S, Bollweg TM, Saboga-Nunes L, Bond E, Sørensen K, Bitzer E, Jordan S, Domanska O, Firnges C, Carvalho GS, Bittlingmayer UH, Levin-Zamir D, Pelikan J, Sahrai D, Lenz A, Wahl P, Thomas M, Kessl F, Pinheiro P (2017). Health literacy in childhood and youth: a systematic review of definitions and models. BMC Public Health.

[9] Jiménez-Pernett J, García-Gutiérrez JF, Bermúdez-Tamayo C, Labry-Lima AO (2010). [Webliography of health for adolescents and young people]. Rev Calid Asist.

[10] Hansen DL, Derry HA, Resnick PJ, Richardson CR (2003). Adolescents searching for health information on the internet: an observational study. J Med Internet Res.

[11] Alnemer KA, Alhuzaim WM, Alnemer AA, Alharbi BB, Bawazir AS, Barayyan OR, Balaraj FK (2015). Are health-related tweets evidence based? Review and analysis of health-related tweets on Twitter. J Med Internet Res.

[12] Ritterband LM, Thorndike FP, Cox DJ, Kovatchev BP, Gonder-Frederick LA (2009). A behavior change model for internet interventions. Ann Behav Med.

[13] Glasgow RE, Klesges LM, Dzewaltowski DA, Bull SS, Estabrooks P (2004). The future of health behavior change research: what is needed to improve translation of research into health promotion practice?. Ann Behav Med.

[14] Bano M, Zowghi D (2015). A systematic review on the relationship between user involvement and system success. Inf Softw Technol.

[15] Dijkstra AA, Voorn P, Berendse HW, Groenewegen HJ, Rozemuller AJ, van de Berg WD, Netherlands Brain Bank (2014). Stage-dependent nigral neuronal loss in incidental Lewy body and Parkinson's disease. Mov Disord.

[16] Rasulov AS, Evstigneeva ZG, Kretovich WL (1978). [Irreversible inactivation of Chlorella glutamine synthetase by urea]. Biokhimiia.

[17] Park E, Kwon M (2018). Health-related internet use by children and adolescents: systematic review. J Med Internet Res.

[18] Moher D, Liberati A, Tetzlaff J, Altman DG (2009). Preferred reporting items for systematic reviews and meta-analyses: the PRISMA statement. PLoS Med.

[19] Sillence E, Briggs P, Harris P, Fishwick L (2006). A framework for understanding trust factors in web-based health advice. Int J Hum Comput Stud.

[20] Hannes K, Lockwood C, Pearson A (2010). A comparative analysis of three online appraisal instruments' ability to assess validity in qualitative research. Qual Health Res.

[21] Campbell R, Pound P, Pope C, Britten N, Pill R, Morgan M, Donovan J (2003). Evaluating meta-ethnography: a synthesis of qualitative research on lay experiences of diabetes and diabetes care. Soc Sci Med.

[22] Reen G, Silber E, Langdon D (2017). Multiple sclerosis patients' understanding and preferences for risks and benefits of disease-modifying drugs: a systematic review. J Neurol Sci.

[23] Ammerlaan JJ, Scholtus LW, Drossaert CH, van Os-Medendorp H, Prakken B, Kruize AA, Bijlsma JJ (2015). Feasibility of a website and a hospital-based online portal for young adults with juvenile idiopathic arthritis: views and experiences of patients. JMIR Res Protoc.

[24] Baulch J, Chester A, Brennan L (2010). Adolescent and parent content preferences and predictors of intention to use an online healthy weight website for adolescents. E-Journal Appl Psychol.

[25] Breakey VR, Warias AV, Ignas DM, White M, Blanchette VS, Stinson JN (2013). The value of usability testing for Internet-based adolescent self-management interventions:. BMC Med Inform Decis Mak.

[26] Breakey VR, Ignas DM, Warias AV, White M, Blanchette VS, Stinson JN (2014). A pilot randomized control trial to evaluate the feasibility of an Internet-based self-management and transitional care program for youth with haemophilia. Haemophilia.

[27] Coyne I, Prizeman G, Sheehan A, Malone H, While AE (2016). An e-health intervention to support the transition of young people with long-term illnesses to adult healthcare services: design and early use. Patient Educ Couns.

[28] Cullen KW, Thompson D, Boushey C, Konzelmann K, Chen T (2013). Evaluation of a web-based program promoting healthy eating and physical activity for adolescents: teen choice: food and fitness. Health Educ Res.

[29] Danielson C, McCauley J, Gros K, Jones A, Barr S, Borkman A, Bryant B, Ruggiero K (2016). SiHLEWeb.com: development and usability testing of an evidence-based HIV prevention website for female African-American adolescents. Health Informatics J.

[30] DeBar L, Dickerson J, Clarke G, Stevens V, Ritenbaugh C, Aickin M (2009). Using a website to build community and enhance outcomes in a group, multi-component intervention promoting healthy diet and exercise in adolescents. J Pediatr Psychol.

[31] Donovan E, Wood M, Frayjo K, Black R, Surette D (2012). A randomized, controlled trial to test the efficacy of an online, parent-based intervention for reducing the risks associated with college-student alcohol use. Addict Behav.

[32] Ercan S (2006). Evaluation of a mental health website for teenagers. Psychiatr Bull.

[33] Hanberger L, Ludvigsson J, Nordfeldt S (2013). Use of a web 2.0 portal to improve education and communication in young patients with families: randomized controlled trial. J Med Internet Res.

[34] Korus M, Cruchley E, Stinson JN, Gold A, Anthony SJ (2015). Usability testing of the Internet program:. Pediatr Transplant.

[35] Long AC, Palermo TM (2009). Brief report: web-based management of adolescent chronic pain: development and usability testing of an online family cognitive behavioral therapy program. J Pediatr Psychol.

[36] McCarthy O, Carswell K, Murray E, Free C, Stevenson F, Bailey JV (2012). What young people want from a sexual health website: design and development of Sexunzipped. J Med Internet Res.

[37] Michaud P, Colom P (2003). Implementation and evaluation of an internet health site for adolescents in Switzerland. J Adolesc Health.

[38] Nicholas D, Fellner K, Frank M, Small M, Hetherington R, Slater R, Daneman D (2012). Evaluation of an online education and support intervention for adolescents with diabetes. Soc Work Health Care.

[39] Nordfeldt S, Hanberger L, Berterö C (2010). Patient and parent views on a Web 2.0 Diabetes Portal--the management tool, the generator, and the gatekeeper: qualitative study. J Med Internet Res.

[40] Radovic A, DeMand A, Gmelin T, Stein B, Miller E (2018). SOVA: design of a stakeholder informed social media website for depressed adolescents and their parents. J Technol Hum Serv.

[41] Radovic A, Gmelin T, Hua J, Long C, Stein BD, Miller E (2018). Supporting Our Valued Adolescents (SOVA), a social media website for adolescents with depression and/or anxiety: technological feasibility, usability, and acceptability study. JMIR Ment Health.

[42] Starling R, Nodulman J, Kong A, Wheeler C, Buller D, Woodall W (2015). Usability testing of an HPV information website for parents and adolescents. Online J Commun Media Technol.

[43] Stinson J, Gupta A, Dupuis F, Dick B, Laverdière C, LeMay S, Sung L, Dettmer E, Gomer S, Lober J, Chan CY (2015). Usability testing of an online self-management program for adolescents with cancer. J Pediatr Oncol Nurs.

[44] Stinson J, McGrath P, Hodnett E, Feldman B, Duffy C, Huber A, Tucker L, Hetherington R, Tse S, Spiegel L, Campillo S, Gill N, White M (2010). Usability testing of an online self-management program for adolescents with juvenile idiopathic arthritis. J Med Internet Res.

[45] Stinson JN, McGrath PJ, Hodnett ED, Feldman BM, Duffy CM, Huber AM, Tucker LB, Hetherington CR, Tse SM, Spiegel LR, Campillo S, Gill NK, White ME (2010). An internet-based self-management program with telephone support for adolescents with arthritis: a pilot randomized controlled trial. J Rheumatol.

[46] Wozney L, Baxter P, Newton A (2015). Usability evaluation with mental health professionals and young people to develop an Internet-based cognitive-behaviour therapy program for adolescents with anxiety disorders. BMC Pediatr.

[47] Gray NJ, Klein JD, Noyce PR, Sesselberg TS, Cantrill JA (2005). The internet: a window on adolescent health literacy. J Adolesc Health.

[48] Ståhl T (2017). How ICT savvy are digital natives actually?. Nord J Digit Lit.

[49] Uncapher MR, Lin L, Rosen LD, Kirkorian HL, Baron NS, Bailey K, Cantor J, Strayer DL, Parsons TD, Wagner AD (2017). Media multitasking and cognitive, psychological, neural, and learning differences. Pediatrics.

[50] Giedd JN (2012). The digital revolution and adolescent brain evolution. J Adolesc Health.

[51] Mills KL, Goddings A, Herting MM, Meuwese R, Blakemore S, Crone EA, Dahl RE, Güroğlu B, Raznahan A, Sowell ER, Tamnes CK (2016). Structural brain development between childhood and adulthood: convergence across four longitudinal samples. Neuroimage.

[52] Göllner LM, Ballhausen N, Kliegel M, Forstmeier S (2017). Delay of gratification, delay discounting and their associations with age, episodic future thinking, and future time perspective. Front Psychol.

[53] Mueller SC, Cromheeke S, Siugzdaite R, Nicolas BC (2017). Evidence for the triadic model of adolescent brain development: cognitive load and task-relevance of emotion differentially affect adolescents and adults. Dev Cogn Neurosci.

[54] Padilla-Walker LM, Carlo G, Memmott-Elison MK (2017). Longitudinal change in adolescents' prosocial behavior toward strangers, friends, and family. J Res Adolesc.

[55] Sebastian C, Viding E, Williams K, Blakemore S (2010). Social brain development and the affective consequences of ostracism in adolescence. Brain Cogn.

[56] Crone E, Konijn E (2018). Media use and brain development during adolescence. Nat Commun.

[57] Kaye S, White M, Lewis I (2017). The use of neurocognitive methods in assessing health communication messages: a systematic review. J Health Psychol.

[58] Happé F, Frith U (2014). Annual research review: towards a developmental neuroscience of atypical social cognition. J Child Psychol Psychiatry.

[59] DuBois D, Lymer E, Gibson B, Desarkar P, Nalder E (2017). Assessing sensory processing dysfunction in adults and adolescents with autism spectrum disorder: a scoping review. Brain Sci.

[60] Ekmekci O (2017). Pediatric multiple sclerosis and cognition: a review of clinical, neuropsychologic, and neuroradiologic features. Behav Neurol.

[61] Imhof M, Schmälzle Ralf, Renner B, Schupp H (2017). How real-life health messages engage our brains: shared processing of effective anti-alcohol videos. Soc Cogn Affect Neurosci.

[62] Raschle NM, Fehlbaum LV, Menks WM, Euler F, Sterzer P, Stadler C (2017). Investigating the neural correlates of emotion–cognition interaction using an affective stroop task. Front Psychol.

[63] Kenny R, Dooley B, Fitzgerald A (2015). Feasibility of. JMIR Ment Health.

[64] Boulos MNK, Maramba I, Wheeler S (2006). Wikis, blogs and podcasts: a new generation of web-based tools for virtual collaborative clinical practice and education. BMC Med Educ.

[65] Reichow B, Gelbar NW, Mouradjian K, Shefcyk A, Smith IC (2014). Characteristics of international websites with information on developmental disabilities. Res Dev Disabil.

[66] Grant N, Rodger S, Hoffmann T (2015). Evaluation of autism-related health information on the web. J Appl Res Intellect Disabil.

[67] Manting EH, Driessen AJ (2000). Escherichia coli translocase: the unravelling of a molecular machine. Mol Microbiol.

[68] Berk L, Berk M, Dodd S, Kelly C, Cvetkovski S, Jorm AF (2013). Evaluation of the acceptability and usefulness of an information website for caregivers of people with bipolar disorder. BMC Med.

[69] Kushalnagar P, Naturale J, Paludneviciene R, Smith S, Werfel E, Doolittle R, Jacobs S, DeCaro J (2015). Health websites: accessibility and usability for American sign language users. Health Commun.

[70] Ryan G, Haroon M, Melvin G (2015). Evaluation of an educational website for parents of children with ADHD. Int J Med Inform.

[71] Scanlan F, Jorm A, Reavley N, Meyer D, Bhar S (2017). Treatment choices for depression: young people's response to a traditional e-health versus a Health 2.0 website. Digit Health.

[72] Hale A, Young V, Grand A, McNulty C (2017). Can gaming increase antibiotic awareness in children? A mixed-methods approach. JMIR Serious Games.

[73] Farrell D, Kostkova P, Weinberg J, Lazareck L, Weerasinghe D, Lecky DM, McNulty CA (2011). Computer games to teach hygiene: an evaluation of the e-Bug junior game. J Antimicrob Chemother.

[74] Sparapani VD, Fels S, Nascimento LC (2017). The value of children's voices for a video game development in the context of type 1 diabetes: focus group study. JMIR Diabetes.

