# Supplementary material for: Usability of Health Information Websites Designed for Adolescents: Systematic Review, Neurodevelopmental Model, and Design Brief
Source: J Med Internet Res. 2019 Apr 23;21(4):e11584. doi: 10.2196/11584 (PMC6658246; doi:10.2196/11584)
Supplement: Multimedia Appendix 1 [file jmir_v21i4e11584_app1.docx]

Multimedia Appendix 1. Quality assessment of included studies (n=25).

| Study (first author, year) | Clear aims | Appropriate methodology | Appropriate research design | Appropriate recruitment strategy | Data collection method | Researcher and participant relationship considered | Ethical issues considered | Rigorous data analysis | Statement of findings | Is the research valuable? |
| --- | --- | --- | --- | --- | --- | --- | --- | --- | --- | --- |
| Ammerlaan 2015 [23] | ✓ | ✓ | ✓ | ✓ | ✓ | ✓ | ✓ | ✓ | ✓ | 🗶 |
| Baulch, 2010 [24] | ✓ | ✓ | ✓ | ✓ | ✓ | 🗶 | ✓ | ✓ | - | ✓ |
| Breakey 2013 [25] | ✓ | ✓ | ✓ | ✓ | ✓ | 🗶 | ✓ | ✓ | ✓ | ✓ |
| Breakey 2014 [26] | ✓ | ✓ | ✓ | ✓ | ✓ | 🗶 | ✓ | ✓ | ✓ | 🗶 |
| Coyne 2016 [27] | ✓ | ✓ | ✓ | ✓ | ✓ | ✓ | ✓ | ✓ | ✓ | ✓ |
| Cullen 2013 [28] | ✓ | ✓ | ✓ | ✓ | ✓ | 🗶 | ✓ | ✓ | ✓ | ✓ |
| Danielson 2016 [29] | ✓ | ✓ | ✓ | ✓ | ✓ | 🗶 | ✓ | ✓ | ✓ | 🗶 |
| Debar 2009 [30] | ✓ | ✓ | ✓ | ✓ | 🗶 | 🗶 | ✓ | ✓ | ✓ | 🗶 |
| Donovan 2012 [31] | ✓ | ✓ | ✓ | ✓ | 🗶 | 🗶 | ✓ | 🗶 | ✓ | 🗶 |
| Ercan 2006 [32] | ✓ | ✓ | ✓ | ✓ | ✓ | 🗶 | ✓ | ✓ | - | ✓ |
| Franck, 2007 [5] | ✓ | ✓ | ✓ | ✓ | ✓ | ✓ | ✓ | ✓ | ✓ | ✓ |
| Hanberger 2013 [33] | ✓ | ✓ | ✓ | ✓ | ✓ | 🗶 | ✓ | 🗶 | - | ✓ |
| Korus 2015 [34] | ✓ | ✓ | ✓ | 🗶 | ✓ | ✓ | ✓ | ✓ | ✓ | 🗶 |
| Long 2009 [35] | ✓ | ✓ | ✓ | ✓ | ✓ | 🗶 | ✓ | 🗶 | ✓ | ✓ |
| McCarthy 2012 [36] | ✓ | ✓ | ✓ | 🗶 | ✓ | ✓ | ✓ | ✓ | ✓ | ✓ |
| Michaud 2003 [37] | ✓ | ✓ | ✓ | 🗶 | ✓ | 🗶 | ✓ | ✓ | - | ✓ |
| Nicholas 2012 [38] | ✓ | ✓ | ✓ | 🗶 | ✓ | ✓ | ✓ | ✓ | ✓ | 🗶 |
| Nordfeldt 2010 [39] | ✓ | ✓ | ✓ | 🗶 | ✓ | - | ✓ | - | ✓ | 🗶 |
| Radovic 2017 [40] | ✓ | ✓ | ✓ | 🗶 | ✓ | ✓ | ✓ | ✓ | ✓ | 🗶 |
| Radovic 2018 [41] | ✓ | ✓ | ✓ | ✓ | ✓ | ✓ | ✓ | ✓ | ✓ | ✓ |
| Starling 2015 [42] | ✓ | ✓ | ✓ | ✓ | ✓ | ✓ | ✓ | - | ✓ | 🗶 |
| Stinson 2010a [43] | ✓ | ✓ | ✓ | ✓ | ✓ | ✓ | ✓ | ✓ | ✓ | ✓ |
| Stinson 2010b [44] | ✓ | ✓ | ✓ | ✓ | 🗶 | - | ✓ | 🗶 | ✓ | ✓ |
| Stinson 2015 [45] | ✓ | ✓ | ✓ | ✓ | ✓ | ✓ | ✓ | ✓ | ✓ | 🗶 |
| Wozney 2015 [46] | ✓ | ✓ | ✓ | ✓ | ✓ | ✓ | ✓ | ✓ | ✓ | 🗶 |

Quality assessed using the Critical Appraisal Skills Programme tool (CASP); all categories marked as either yes (✓), no (🗶) or can't tell/unclear (-)
