# Supplementary material for: Usability of Health Information Websites Designed for Adolescents: Systematic Review, Neurodevelopmental Model, and Design Brief
Source: J Med Internet Res. 2019 Apr 23;21(4):e11584. doi: 10.2196/11584 (PMC6658246; doi:10.2196/11584)
Supplement: Multimedia Appendix 2 [file jmir_v21i4e11584_app2.docx]

Multimedia Appendix 2**:** Website characteristics and patient feedback about website usability (n=25).

| Study (first author, year) | Website name or URL  (website status) | Topic of website | Website launched | Website features | Method of evaluation | Positive feedback | Negative feedback |
| --- | --- | --- | --- | --- | --- | --- | --- |
| Ammerlaan 2015 [23] | Jong en Reuma (currently online) | Juvenile Idiopathic Arthritis | Not specified | Log-in feature, communication with a specialist, access to medical record, self-monitoring (e.g. online diary) | Interviews | Preferred features:  Website design, easy to read, well-targeted to young adults, videos, life stories of other patients  Features wanted:  Facility to make online appointments, access to x-rays, printing forms for blood collection, opening hours of specialists  Most used tool:  Access to personal medical records | Features not preferred:  Log-in code too long and complex, access to medical records overwhelming,  Least used tool:  Communication with specialist (n=4)  Self-monitoring (n=4) |
| Baulch, 2010 [24] | Not specified | Weight management | Not specified | Monitoring, reward ideas, links to additional ideas, charts to plot daily activity, colour, interactive, discussion board, health professional to answer questions | Modified TAM scale (12-item measure of usefulness and ease of use on 1-7 Likert scale)  Survey | Perceived usefulness (M=5.04, SD=0.98), perceived ease of use (M=5.48, SD=0.98), intention to use (M=4.82, SD=1.48)  Most preferred features: Links to additional ideas (97%), charts (84%), email feedback (82%), login (79%), links to other websites (76%), phone support (61%), discussion board (57%), chat room (52%)  Additional preferred features: Games, success stories, motivation quotes | Least preferred feature:  Online journal (37%) |
| Breakey 2013 [25] | Teens taking charge: Managing haemophilia online  (currently online) | Haemophilia | Not specified | 80 web pages of content, images, interactive animations, quizzes, glossary, self-management strategies | Think-aloud procedure  Interviews | Preferred features:  Colour, chunking of information, images, animations, interview of peers with condition | Features not preferred:  Videos too dark (n=3), Venn diagrams difficult to comprehend |
| Breakey 2014 [26] | Teens taking charge: Managing haemophilia online  (currently online) | Haemophilia | Not specified | 80 web pages of content, images, interactive animations, quizzes, glossary, self-management strategies | Survey | Preferred features:  Easy to use, videos, animations, relaxation exercises | Least used feature:  Internet forum |
| Coyne 2016 [27] | www.SteppingUP.ie (currently online) | Transition from paediatric to adult healthcare | 2013 | Home page, top-tips section, 9 video testimonials, FAQs, essential reading, external links, photo gallery | Think-aloud procedure | Preferred features:  Good layout, easy navigation, testimonials, short length videos (2-5 minutes), age-appropriate, colourful  Features wanted:  Downloadable information sheets, visual images of clinics and hospitals | Features not preferred:  Pages too long, too much information, bright orange colour, picture of young person considered cheesy, did not understand meaning of FAQ |
| Cullen 2013 [28] | Teen choice: food and fitness  (not found) | Physical activity and diet | Not specified | Log-in feature, healthy eating calculator, goal setting, 12 short role-model video stories, Did you know section, blog, track goal progress (diary), print goal sheet | Survey | Preferred features:  Healthy eating calculator (89%), Goal setting (91%), Did you know section (88%) | Least used features:  Diary used three or more times (33%) |
| Danielson 2016 [29] | SiHLEweb.com (currently online) | HIV prevention | Not specified | Subsections, videos, photos of women, interactive quizzes | Survey  Interviews | Preferred features:  Videos, age-appropriate, interactive activities and quizzes, pictures of other females | Features not preferred:  Videos too long, mixed reviews on navigation (some found it easy to navigate and some found it difficult to navigate) |
| Debar 2009 [30] | The Youth, Osteoporosis and Understanding Total Health Project  (not found) | Physical activity and diet | Not specified | Log-in feature, bulletin board to communicate with peers and staff, special form to ask questions to staff for confidential reply, forum to post anonymous questions, handouts, links to external content, hot tips section providing short summaries, photos of staff, quizzes, options to win prizes, incentive points, My progress page | Survey  Website visits | Most preferred features:  Incentive points system (50%), Learning new information (37%)  Most visited pages:  Fun stuff such as quizzes (35%), Social networking features (29%) Scrapbook (24%) | Least preferred features:  Information not regularly updated (30%)  Least visited pages:  Hot tips summaries (8%), additional resources (4%) |
| Donovan 2012 [31] | Self-management program for migraines  (not found) | Migraines | Not specified | Quizzes, audio and video-based tools, social networking, virtual toolbox of coping strategies, headache diay | Survey | Features preferred:  Ask an expert feature, video-based content  Features wanted:  Library of content |  |
| Ercan 2006 [32] | Ru-ok.com (not found) | General  Includes: mental health, eating problems, drugs and alcohol | 2003 | interactive stories, cartoons about depression, games | Survey | Preferred features:  Graphics/pictures (86.6%), site customisation (85.1%), games (76.9%), interactive stories (83.1%) |  |
| Franck, 2007 [5] | www.childrenfirst.nhs.uk (not online) | General  Includes: body, chronic conditions, disabilities, tests, treatments | 2001 | Sections dedicated to children, teens and families section | Analysis of 30 min website navigation  Survey  Informal discussion | Navigation:  45% went to older adolescent section before going to section suitable for age  Preferred features:  Pictures, games and animations, real stories  Features wanted:  Positive recovery stories | Features not preferred:  Gender imbalanced pages, too much text in children section, the cartoon characters used, real stories section too negative, , older adolescent page considered dull and boring, disagreement for pages too crowded or too plain |
| Hanberger 2013 [33] | Diabit web 2.0 portal (not found) | Diabetes | 2006 | Social networking, discussion board, local practitioners details, information about local activities, new research, questions and answers, photos of staff, education videos, date of last update and people who wrote webpage | Website visits | Most visited pages:  Home page (10%), stories section (5.3%), blogs (7.5%), questions and answers (1.8%), videos (2.7%), discussion board (1.2%) | Least visited page:  External links (0%) |
| Korus 2015 [34] | Teens taking charge: Managing my transplant online  (currently online) | Transplant | Not specified | Videos, colourful | Think-aloud procedure  Semi-structured interviews | Preferred features:  Visually-appealing with lots of colour, video testimonials  Features wanted:  Hyperlinks to other pages within module, a search box, drop-down menus, more colour, more pictures and graphics, a discussion forum, music, interactive qualities | Features not preferred:  Mixed reviews about navigation (some found it easy to navigate and others found it difficult to navigate) |
| Long 2009 [35] | Web-MAP program (not found) | Chronic pain | Not specified | Video interviews of peers with condition, relaxation audio clips, 200 content pages with graphics, log-in, goal setting, interactive, questions and answers | Stage 1:  Survey for users with past history of chronic pain (1-5 Likert scale)  Stage 2:  Survey for users with current chronic pain; 1-5 Likert scale) | Stage 1  Perceived ease of use: (M=4.40, SD=0.55)  Stage 2  Perceived ease of use: (Mean=4.50, SD=0.64)  Preferred features:  Easy to navigate, video, audio, personalisation | Stage 1:  Features not preferred:  Difficult to understand images, lengthy content  Stage 2:  Features not preferred:  Some images, long load times |
| McCarthy 2012 [36] | Sexunzipped website (not found) | Sexual health | Not specified | Interactive quizzes, short interactive activities, peer-to-peer exchange of views  (website deigned after user feedback) | Focus groups | Preferred features:  Social interaction, anonymous, videos about peers discussing real stories, commenting on videos, dramatic story format, images of people, images of scenarios, images about specific sexual health issues, easy to understand website logo,, clear and memorable website name, weekly update, interactive activities | Features not preferred:  Too much text, disagreement on colour (some users preferred bold colours and others preferred neutral tones) |
| Michaud 2003 [37] | [www.ciao.ch](http://www.ciao.ch) (currently online) | General  Includes: drug, alcohol, contraception, sleep patterns | 1993 | Emailed questions answered by professionals, addresses of professional institutions, list of previously asked questions | Surveys  Website visits | Preferred features:  Structured answers to questions, list of other questions, addresses of other institutions  Most visited page:  Questions and answers (82%) |  |
| Nicholas 2012 [38] | Online modules on AboutKidsHealth information website (not found) | Diabetes | Not specified | Not specified | Interviews | Preferred features:  Discussion topics on forum, anonymity  Features wanted:  Personalisation, instant-messaging |  |
| Nordfeldt 2010 [39] | Diabit web 2.0 portal (not found) | Diabetes | 2006 | Social networking, discussion board, local practitioners details, information about local activities, new research, questions and answers, photos of staff, educational videos, date of last update and people who wrote webpage | Qualitative essays | Preferred features:  Facts, simple layout, easy to use and log-in | Features not preferred:  Difficult chatting feature, difficult log-in as password too hard to recover |
| Radovic 2017 [40] | sova.pitt.edu (currently online) | Depression | Not specified | Blog posts, Links to external resources, personalisation, questions posed for user discussion | Phase 1: interviews  Phase 2: Think-aloud procedure and System Usability Scale (1-5 Likert scale) | Phase 1:  Most preferred features: online peer support, blogs, moderator to avoid sharing incorrect information with other peers, anonymity.  Phase 2:  100% completed all think-aloud tasks  Usability score mean = 4.5 (sd=.31) | Phase 2:  Least preferred feature:  Wiki tool to contribute to a story |
| Radovic 2018 [41] | sova.pitt.edu (currently online) | Depression | Not specified | Blog posts, links to external resources, personalisation, questions posed for user discussion | System usability scale (1-5 Likert scale)  Survey  Interview | Preferred features:  Age appropriate format, positive atmosphere, interactive, regular new content, positive stories, anonymity  Features wanted:  Social interaction, interactive  Rating of user friendliness:  50% users thought user friendly of site was good | Features not preferred:  Structured communication on forum, log-in |
| Starling 2015 [42] | GoHealthyGirls website (not found) | HPV | Not specified | Interactive quiz show game, texting stimulation, FAQ section | Analysis of navigation  System usability scale (Rating of user friendliness, 1-7 Likert scale)  Survey | Preferred features:  Easy to use, website functions  Rating of user friendliness: (M=6.0)  Features wanted:  Clear title | Features not preferred:  Sound levels of videos too high |
| Stinson 2010a [43] | Teens taking charge: Managing arthritis online  (currently online) | Juvenile Idiopathic Arthritis | Not specified | 310 content pages, animations, images, videos of peers with medical condition, written stories, discussion boards, surveys, quizzes, glossary of medical terms, relaxation audio clips, visual imagery audio clips, printable PDF information forms for teachers, journal for symptom tracking and weekly goals, ask the expert features, personalisation | Analysis of 30-45min navigation  Think-aloud  Semi-structured interviews | Preferred features:  Animations, chunking of text, layout, simple website, up-to-date, age-appropriate, glossary of medical terms, ask the expert, pdf forms, audio clips, journal, discussion boards  Features wanted:  Chunking of texts using graphics and animations, labels on medical diagrams, important information on top of page | Navigation errors:  Overall navigation errors (10%)  Presentation error (26%)  Control usage of animations and videos error (42%)  Features not preferred:  Teenager looking sad, control buttons on animations and videos not easy to see |
| Stinson 2010b [44] | Teens taking charge: Managing arthritis online  (currently online) | Juvenile Idiopathic Arthritis | Not specified | 310 content pages, animations, images, videos of peer with medical condition, written stories, discussion boards, surveys, quizzes, glossary of medical terms, relaxation audio clips, visual imagery audio clips, printable PDF information forms for teachers, journal for symptom tracking and weekly goals, ask the expert features, personalisation | Survey | Preferred features:  Easy to use, video, relaxation audio guide, visual imagery audio guide, personalisation |  |
| Stinson 2015 [45] | Teens taking charge: cancer  (in development) | Cancer | Not specified | 200 contents pages, animations, images, videos, discussion boards, surveys and interactive forms, log-in required | Analysis of 30-40min navigation  Think-aloud  Semi-structured interviews | Preferred features:  Colour scheme, sections and subsections making it easy to locate information, bright colours, graffiti-style wallpaper, simple words with not too much jargon, videos from experts, glossary of terms, interactive components, video clips of other adolescents  Features wanted:  Font and appearance to take up more room, search bar at the top of the page | Features not preferred:  Too much blank 'white' space |
| Wozney 2015 [46] | Breathe program (not found) | Anxiety | Not specified | Personalisation (Email reminders, pre-populating answers, notifications), videos, log-in, check-in & check-out, homework activities | Interviews | Preferred features:  Introductory animation video, personalisation, comic book style, videos, images, graphics, animations, logos  Features wanted:  More videos and images needed | Features not preferred:  Slow page loading, no chunking, not visually pleasing |
